# Targeting the Fcμ-receptor in chronic lymphocytic leukemia with a novel IgM-derived antibody-drug conjugate

**DOI:** 10.1186/2051-1426-1-S1-P136

**Published:** 2013-11-07

**Authors:** Martin Skarzynski, Bérengère Vire, Joshua D Thomas, Christopher G Nelson, Alexandre David, Georg Aue, Terrence R Burke, Christoph Rader, Adrian Wiestner

**Affiliations:** 1 Hematology Branch, National Heart, Lung, and Blood Institute, Bethesda, MD, USA; 2 Chemical Biology Laboratory, Molecular Discovery Program, Frederick National Laboratory for Cancer Research, Center for Cancer Research, National Cancer Institute, Frederick, MD, USA; 3 Laboratory of Viral Diseases, National Institute of Allergy and Infectious Diseases, Bethesda, MD, USA; 4 Experimental Transplantation and Immunology Branch, Center for Cancer Research, National Cancer Institute, Bethesda, MD, USA; 5 Department of Cancer Biology and Department of Molecular Therapeutics, The Scripps Research Institute, Scripps Florida, Jupiter, FL, USA

## 

Fc-receptors (FcR) are widely expressed on cells of the immune system. FcμR is a transmembrane protein with an extracellular Ig-like domain homologous to the FcR for both IgA and IgM (Fcα/μR) and the polymeric Ig receptor (pIgR). FcμR is expressed on CD19+B cells, CD4+/CD8+ T cells, and CD56+/CD3- NK cells. In addition, several groups have reported that FcμR is overexpressed in chronic lymphocytic leukemia (CLL) cells. Using immunofluorescence staining, we found that FcμR can rapidly uptake IgM, internalize it in specific vesicles and transport it through the endocytic pathway to the lysosomal compartment. Interestingly, aggregation of FcμR with IgM leads to rapid internalization of IgM (>80% internalized within 5 minutes) whereas mAb bound FcμR is not internalized. Overexpression on CLL cells and rapid internalization of FcμR represents a potential means of selectively delivering a cytotoxic agent into malignant cells. To this end, we engineered a protein scaffold derived from the CH2-CH3-CH4 IgM constant regions with a C-terminal selenocysteine that allows covalent conjugation of drugs or toxins to the protein scaffold. We verified that the scaffold also binds FcμR, is rapidly internalized and has a serum circulatory half-life comparable to IgM (~18hrs) in NOD/SCID/IL-2Rγnull (NSG) mice. We then demonstrated that the scaffold, when conjugated to a cytotoxic small molecule, kills malignant B cells, but not normal T cells, from CLL patients in vitro and in NSG mice. These findings indicate that the rapid internalization of IgM-FcμR complexes can be exploited for therapeutic purposes. Taken together, IgM-derived protein scaffold antibody-drug conjugates appear as promising treatment modalities for CLL and possibly other malignancies.

